# AWAKEN-Ing a New Frontier in Neonatal Nephrology

**DOI:** 10.3389/fped.2020.00021

**Published:** 2020-02-07

**Authors:** David Joseph Askenazi

**Affiliations:** ^1^Section of Pediatric Nephrology, Department of Pediatrics, University of Alabama at Birmingham, Birmingham, AL, United States; ^2^Pediatric and Infant Center for Acute Nephrology, Children's of Alabama, Birmingham, AL, United States

**Keywords:** acute kidney injury, neonate, acute renal failure, collaborative, epidemiology, outcomes, survival

## Abstract

In 2013, literature about the epidemiology of neonatal acute kidney injury (AKI) was limited to primarily retrospective, single center studies that suggested that AKI was common and that those with AKI had higher rates of mortality. We developed a 24-center retrospective cohort of neonates admitted to the NICU between January 1 and March 31, 2014. Analysis of the Assessment of Worldwide Acute Kidney Epidemiology in Neonates (AWAKEN) cohort, has allowed us to describe the prevalence, risk factors and impact of neonatal AKI for different gestational age cohorts. The ample sample size allows us to provide convincing data to show that those with AKI have an increase *independent* higher odds of death and prolonged hospitalization time (1). This data mirrors similar studies in pediatric (2) and adult (3) critically ill populations which collectively suggest that patients do not just die with AKI, but instead, AKI is directly linked to hard clinical outcomes. This study has allowed us to answer multiple other questions in the field which has expanded our understanding of the risk factors, complications, impact of fluid overload, the definition of neonatal AKI and suggests interventions for improving outcomes. Furthermore, this project brought together neonatologist and nephrologist within and across centers. Finally, the AWAKEN project has enabled us to build relationships and infrastructure that has launched the Neonatal Kidney Collaborative http://babykidney.org/ on its way to accomplish its stated mission to improve the health of newborns with or at risk for kidney disease through multidisciplinary collaborative research, advocacy, and education.

On April 9, 2013, the National Institute of Health sponsored a workshop in Washington DC with the following objectives (1) review the state-of-the-art knowledge of acute kidney injury (AKI) in neonates; and (2) determine the feasibility of studying this group in an organized prospective manner. This conference brought together experts from the fields of pediatric nephrology, neonatology, general pediatrics, industry, and professional organizations to get a broad perspective on the issues to be considered. Two white papers were published. The first, reports a framework whereby the scientific community can answer critical questions about how and when to evaluate neonates at risk for chronic kidney disease (4). The second, focused on the definition of neonatal AKI (5). Furthermore, this meeting solidified the need to develop a multi-center, multi-disciplinary, neonatal kidney collaborative.

Up until this time, the field of neonatal AKI was limited to small single center studies. The use of the staged AKI criteria had only just begun to be used in neonatal studies. Using these staged AKI definitions, small single-center neonatal studies of very low birth weight neonates (6–11), term asphyxiated infants (12–15), those who underwent extra-corporeal membrane oxygenation (16–18), and cardiac pulmonary bypass surgery (19–22) had rates of AKI between 10 and 83%. Consistently in these manuscripts, those with AKI had higher mortality than those without AKI; however, due to their relative small sample size, it was difficult to surmise the independent impact of AKI on survival after accounting for confounders.

At the American Society of Nephrology meeting in Atlanta Georgia on November 7, 2013, a group of pediatric nephrologist agreed to form a collaborative (27 and 5/7 weeks after conception at the NIH Neonatal AKI workshop). The following Spring at the Pediatric Academic Society in Vancouver, Canada we had our first official Neonatal Kidney Collaborative meeting. At this meeting interested neonatologist and nephrologist began to develop the mission, vision, strategy, and necessary infrastructure for sustained collaboration. Shortly thereafter, researchers who attended the NIH workshop, and those who were doing single-center neonatal AKI studies joined the group. The only criteria to join was a commitment to participate in a retrospective multi-center study, and an identified neonatology and pediatric nephrologist at the institution willing to work together on the project. Our short-term goals were simple: first, to develop an infrastructure for communication and knowledge acquisition; second, to perform a multi-center epidemiology study that would improve our understanding of the practice patterns, incidence, and outcome in neonates spanning the gestational age spectrum who were critically ill. Our long-term goal was to improve the short and long-term outcomes for neonates at risk for kidney disease.

With commitments from 24 centers, the Neonatal Kidney Collaborative worked to develop the questions, the data forms, the database and the committee infrastructure for our inaugural project, the Assessment of Worldwide Acute Kidney Epidemiology in Neonates (AWAKEN)^1^. This acronym symbolizes our intention to “wake up” the community to the need to better understand neonatal kidney disease. Fortunately we had support from many. We partnered with Dr. Stuart Goldstein, who had recently completed the collection of data from children admitted to the pediatric intensive care unit in a study called Assessment of Worldwide Acute kidney injury and Renal Angina Epidemiology (AWARE). Leveraging these resources, we developed a web based data entry system for the AWAKEN study.

We outlined the most important questions we could answer through a multi-center retrospective study. One of the most important decisions we had to make up front was to determining the inclusion and exclusion criteria. Recognizing that many babies who are only in the NICU for a short duration (i.e., transient tachypnea of the newborn) do not get assessed for kidney disease, we chose to only look at infants who received intravenous fluids for more than 48 h as a key inclusion criteria. In addition we chose to only include infants who were admitted to our NICU's within the first 2 weeks of life, and we excluded those who had severe congenital heart disease requiring heart surgery within the first perinatal week, those who died within 48 h (as we could not assign them to having AKI or not), those with lethal chromosome anomalies, and those with severe bilateral congenital kidney disease. Of the infants who were admitted to the hospital, about 50% met inclusion/exclusion criteria. Thus, the AWAKEN study should not be generalized to all neonates, nor all who are admitted to the NICU; instead, the AWAKEN study can be generalized only to sick infants who need extensive support beyond 48 h after birth. The methods for the study were published prior to data analysis (23).

As of December 2019, we have published 13 original manuscripts from this cohort which we summarize in Table 1. We are planning additional manuscripts as we continue to pose and test specific hypotheses. All manuscripts have neonatology and nephrology representation, and most have a neonatologist and nephrologist as first and last authors pairs. Most first-author for these manuscripts have been led by early academic investigators, medical students, and fellows. The first four sets of questions (epidemiology, risk factors, fluid balance, and definition) were determined prior to the data abstraction and were led by the Neonatal Kidney Collaborative steering committee. The rest of the manuscripts below were developed via the secondary analysis manuscript process.

**Table 1:** Original manuscript published as of January 2020 from the AWAKEN Study.

**Author**	**Journal**	**Article name**	**Summary of findings**	**DOI**
Starr et al.	American Journal of Perinatology, November 2019	Acute Kidney Injury and Bronchopulmonary Dysplasia in Premature Neonates Born <32 Weeks' Gestation.	Moderate or severe broncho-pulmonary dysplasia (BPD) occurred in 214 of 546 (39%) infants, while death occurred in 32 of 546 (6%); the composite of moderate or severe BPD/death occurred in 246 of 546 (45%). For infants born ≤ 29 weeks of gestation, the adjusted odds ratio (OR) of AKI and the primary outcome was 1.15 [95% confidence interval (CI) = 0.47–2.86; *p* = 0.76]. Infants born between 29 and 32 weeks of gestation with AKI had four-fold higher odds of moderate or severe BPD/death that remained after controlling for multiple factors (adjusted OR = 4.21, 95% CI: 2.07–8.61; *p* < 0.001). Infants born between 29 and 32 weeks of gestation with AKI had four-fold higher odds of moderate or severe BPD/death that remained after controlling for multiple factors (adjusted *OR* = 4.21, 95% CI: 2.07–8.61; *p* < 0.001).	doi: 10.1055/s-0039-3400311
Starr et al.	American Journal of Perinatology, November 2019	Acute Kidney Injury is Associated with Poor Lung Outcomes in Infants Born >32 Weeks' Gestation.	Chronic Lund Disease (CLD) occurred in 82/1,348 (6.1%) infants, while death occurred in 22/1,348 (1.6%); the composite of CLD/death occurred in 104/1,348 (7.7%). Infants with AKI had an almost five-fold increased odds of CLD/death, which remained after controlling for GA, maternal polyhydramnios, multiple gestations, 5-min Apgar's score, intubation, and hypoxic-ischemic encephalopathy [adjusted odds ratio (OR) = 4.9, 95% confidence interval (CI): 3.2–7.4; *p* < 0.0001]. Infants with AKI required longer duration of respiratory support (count ratio = 1.59, 95% CI: 1.14–2.23, *p* = 0.003) and oxygen (count ratio = 1.43, 95% CI: 1.22–1.68, *p* < 0.0001) compared with those without AKI.	doi: 10.1055/s-0039-1698836
Selewski et al.	Pediatric Research, September 2019	The impact of fluid balance on outcomes in premature neonates: a report from the AWAKEN study group.	One hundred and forty-nine (14.8%) were on mechanical ventilation (MV) at post-natal day 7. The median peak Fluid Balance (FB) was 0% (IQR: −2.9, 2) and occurred on post-natal day 2 (IQR: 1,5). Multivariable models showed that the peak FB (aOR 1.14, 95% CI 1.10–1.19), lowest FB in first post-natal week (aOR 1.12, 95% CI 1.07–1.16), and FB on post-natal day 7 (aOR 1.10, 95% CI 1.06–1.13) were independently associated with MV on post-natal day 7. In a similar analysis, a negative FB at post-natal day 7 protected against the need for MV at post-natal day 7 (aOR 0.21, 95% CI 0.12–0.35).	doi: 10.1038/s41390-019-0579-1
Stoops et al.	Neonatology, August 2019	The Association of Intraventricular Hemorrhage and Acute Kidney Injury in Premature Infants from the Assessment of the Worldwide Acute Kidney Injury Epidemiology in Neonates (AWAKEN) Study.	AKI was documented in 22.2% (183/825) of infants and Intraventricular hemorrhage (IVH) in 14.3% (118/825). Infants with AKI (*n* = 183) were more likely to have IVH (26.8%, 49/183) than those without AKI (*n*= 642) who had IVH (10.7%, 69/642, *p* < 0.0001). After controlling for 5-min Apgar score, vasopressor support within the first week of age, and gestational age, infants with AKI had 1.6 times higher adjusted odds to develop any grade IVH (95% CI 1.04–2.56). Furthermore, infants of gestational age of 22–28 weeks had 1.9 times higher adjusted odds to develop IVH (OR 1.87, 95% CI 1.08–3.23).	doi: 10.1159/000501708
Charlton et al.	Clinical Journal of American Society of Nephrology, February 2019	Incidence and Risk Factors of Early Onset Neonatal AKI.	In over 2,000 patients, early AKI (≤7 days) occurred in 21% of neonates. Infants with early AKI had higher risk of death (aOR 2.8, 95% CI 1.7–4.7) and longer length of stay (7.3 days, 95% CI 4.7–10). Risk factors for early AKI are: outborn delivery; resuscitation with epinephrine; admission diagnosis of hyperbilirubinemia, inborn errors of metabolism, or surgical need; frequent kidney function surveillance; and admission to a children's hospital. Protective factors were: multiple gestations, cesarean section, and exposures to antimicrobials, methylxanthines, diuretics, and vasopressors.	doi: 10.2215/CJN.03670318
Harer et al.	JAMA Pediatrics, April 2018	Association Between Early Caffeine Citrate Administration and Risk of Acute Kidney Injury in Preterm Neonates: Results from the AWAKEN Study.	Of 675 preterm infants ≤ 33 weeks, AKI occurred less frequently in neonates who received caffeine than those who did not [50 of 447 (11.2%) vs. 72 of 228 (31.6%), *P* < 0.01]. After multivariable adjustment, the number needed to treat to prevent one case of AKI was 4.3 and those receiving caffeine were less likely to develop high grade AKI (stage 2 or 3, OR 0.20, 95% CI 0.12–0.34).	doi: 10.1001/jamapediatrics.2018.0322
Kraut et al.	Pediatric Research, May 2018	Incidence of neonatal hypertension from a large multicenter study [Assessment of the Worldwide Acute Kidney Injury Epidemiology in Neonates (AWAKEN)].	Of over 2,000 infants, hypertension was documented in 1.8% and an additional 3.7% were defined as having undiagnosed hypertension. Hypertension was associated with a diagnosis of AKI and other risk factors for HTN were hyperbilirubinemia, Caucasian race, outborn, vaginal delivery, and congenital heart disease. Protective factors were small for gestational age, multiple gestation, and maternal betamethasone.	doi: 10.1038/s41390-018-0018-8
Kirkley et al.	Pediatric Research, August 2018	Acute kidney injury in neonatal encephalopathy: an evaluation of the AWAKEN database.	Of 113 patients with neonatal encephalopathy, 41.6% developed AKI. Risk factors for AKI were outborn, Intrauterine growth retardation, and presence of meconium at delivery. AKI resulted in longer hospital stays (8.5 days, 95% CI 0.79–16.2).	doi: 10.1007/s00467-018-4068-2
Selewski et al.	Pediatric Research, September 2018	The impact of fluid balance on outcomes in critically ill near term/term neonates: a report from the AWAKEN study group.	The median peak fluid balance was 1.0% and occurred on post-natal day 3. Multivariable models showed the peak fluid balance, lowest fluid balance in 1st post-natal week, and fluid balance on post-natal day 7 were independently associated with need for mechanical ventilation on post-natal day 7.	doi: 10.1038/s41390-018-0183-9
Askenazi et al.	Pediatric Research, December 2018	Optimizing the AKI definition during the first post-natal week using Assessment of Worldwide Acute Kidney Injury Epidemiology in Neonates (AWAKEN) cohort.	The absolute rise in serum creatinine of 0.3 mg/dL outperformed a ≥50% rise in serum creatinine during the first week of life for predicting mortality. The optimal serum creatinine thresholds to predict AUC and specificity were ≥0.3 and ≥0.6 mg/dL for ≤ 29 weeks gestational age and ≥0.1 and ≥0.3 mg/dL for >29 week gestational age. The maximum serum creatinine value provides great specificity.	doi: 10.1038/s41390-018-0249-8
Charlton et al.	Pediatric Research, December 2018	Late onset neonatal acute kidney injury: results from the AWAKEN study.	*n* over 2,000 patients, late AKI (>7 days after birth) occurred in 9% of neonates. Infants with late AKI had increased risk of death (aOR 2.1, *p* = 0.02) and longer length of stay (21.9, *p* < 0.001). Risk factors for late AKI are: intubation, oligo- and polyhydramnios, mild-moderate renal anomalies, admission diagnoses of congenital heart disease, necrotizing enterocolitis, surgical need, exposure to diuretics, vasopressors, and NSAIDs, discharge diagnoses of patent ductus arteriosus, necrotizing enterocolitis, sepsis, and urinary tract infection.	doi: 10.1038/s41390-018-0255-x
Jetton et al.	Lancet Child Adolescent Health. September 2017	Incidence and outcomes of neonatal acute kidney injury (AWAKEN): multicenter, multinational, observational cohort study.	In over 2,000 infants admitted to the NICU on IVF for at least 48 h, 30% developed AKI based on the neonatal KDIGO definition. AKI varies by gestational age at birth: 48% for those born 22–29 weeks, 18% for 29–35 weeks, and 37% for babies ≥36 weeks. Babies with AKI have higher mortality (OR 4.6, 95% CI 2.5–8.3) and longer length of hospital stay (8.8 days, 95% CI 6.1–11.5) after adjusting for multiple confounding factors.	doi: 10.1016/S2352-4642(17)30069-X
Jetton et al.	Frontiers in Pediatrics, July 2016	Assessment of Worldwide Acute Kidney Injury Epidemiology in Neonates: Design of a Retrospective Cohort Study.	Describes the formation of the NKC and establishment of the AWAKEN cohort and database—the largest most inclusive neonatal AKI study to date.	doi: 10.3389/fped.2016.00068

The primary hypothesis for AWAKEN was that AKI was independently associated with mortality after controlling for numerous confounders. Using the Neonatal KDIGO AKI definition, we found that ~30% of the cohort had at least one episode of AKI. Interestingly, the incidence differed across the gestational age in a “U” distribution. The incidence of AKI was 43% in those <29 weeks GA, 18% in those between 29 and 36 weeks GA, and 37% in the those >36 weeks GA. Of the 605 infants with AKI, 59 (9.7%) died compared to only 20/1,417 (1.4%) who did not have AKI. Even after controlling for numerous confounders known to be associated with neonatal mortality, the adjusted OR for death in those that had AKI was 4.6 times higher the odds of death in those who did not have AKI. Furthermore, those with AKI had an adjusted 8.8 more hospital days compared to those without AKI (1). These relationships held true when we explored subsets of patients categorized by gestational age.

One of the unique parts of this study was that for the first time, we are able to compare the risk factors of AKI in neonates of different GA ranges, and in different time points of the hospital course. We published on these risk factor of early neonatal AKI (first perinatal week) where we showed how perinatal risk factors (maternal and infant demographics, APGAR scores, perinatal medications) are closely associated with AKI (24). Next we reported the risk factors of late AKI (after the post-natal week). After the first week, the perinatal factors are less as important in predicting AKI, but a previous episode of AKI, sepsis, surgery, and nephrotoxin medications are risk factors for AKI (25). For both of these timeframes, we describe the risk factors by different gestational age groups. Currently, we are also describing how anemia, hypoalbuminemia, and dysnatremias are associated with early neonatal AKI (presented as abstracts—not yet in press).

The impact of fluid balance in critical illness is one of the most important questions in critical care nephrology. Besides a few reports on neonates who required extra-corporeal membrane oxygenation and those who had cardio-pulmonary bypass surgery, there is a paucity of data on the impact of fluid balance and neonatal outcomes. The AWAKEN study allows us to explore these relationships as we show that different fluid balance parameters during the first perinatal week predict the need for mechanical ventilation at 7 days, even after controlling for multiple potential confounders in premature neonates (26) and in near-term/term neonates (27).

One of the most challenging aspects to the evaluation and clinical research on neonatal AKI is the complexity of interpreting the normal SCr patterns seen during the first perinatal weeks and the pragmatic approach to defining neonatal AKI. We use the AWAKEN database to show that different GA groups have different optimal SCr cutoffs at different timepoints after birth to predict mortality. In addition, we show that the addition of a percent rise in SCr does not add any important information to an absolute SCr rise in the ability to predict mortality. This has allowed us to propose a framework for future investigations in understanding how to diagnose neonatal AKI, which will need to be tested in other large clinical cohorts (28).

In a manuscript published in JAMA-Peds, we show that despite the fact that infants who received caffeine (commonly done to keep infants from needing to get intubated) were sicker, those who received caffeine had a much lower adjusted odds of developing AKI than those who were not exposed to caffeine (number needed to treat = 4.3) (29). Other ancillary studies include a report the association of AKI and Hypertension (30), a study showing the association between AKI and mortality in those with severe neonatal encephalopathy (31), the association of AKI and Intra-ventricular hemorrhage (32), the association of AKI and Chronic Lung Disease in premature (33) and near term/term infants (34).

## Implications for the Future

The AWAKEN study has allowed us to answer multiple previously unanswered questions, and has “AWAKEN'ed” the field of Neonatal Kidney Disease. We have shown that AKI is very common in sick critically ill neonates, and those who have AKI have a much higher mortality risk than those without AKI. Thus, it is no longer acceptable for the medical community to say that neonatal AKI is rare and carries no sequalae. We have identified that caffeine may prevent AKI, which may have implications not only in neonates but for other populations. Furthermore, we have shown a wide disparity in evaluating for AKI using SCr, and not surprisingly, those centers who measure SCr often have much higher rates of AKI, suggesting a wide practice variation. We have described the potential consequences of impaired kidney function in the neonate (impaired fluid balance, blood pressure control) and its associations with chronic lung disease and intra-ventricular hemorrhage. Finally, this dataset allows centers to compare their current practice to the group as a whole as we provided center-specific data in relation to the AWAKEN cohort collectively.

Importantly, we have supported the ability for medical students, residents, fellows and young attendings to lead manuscripts, and participate in the project. We hope that this experience will stimulate their academic careers with an emphasis on neonatal nephrology, thereby enriching the field with talented, young academicians with strong mentors from the group. Importantly, AWAKEN has provided neonatologist and nephrologist interested in neonatal nephrology an opportunity to problem-solve, study, interpret data, and share ideas together. Finally, the answer to these questions stimulates researchers to ask the next set of questions and motivation us to improve outcomes in this vulnerable population.

## Author Contributions

The author confirms being the sole contributor of this work and has approved it for publication.

### Conflict of Interest

The author declares that the research was conducted in the absence of any commercial or financial relationships that could be construed as a potential conflict of interest.

## Footnotes

^1^Supplementary Presentation 1 is a slide-deck used as part of a presentation on AWAKEN presented at the 3rd pAKI meeting in Cincinnati Ohio in October 2018.

## References

[1] Jetton JG Boohaker LJ Sethi SK Wazir S Rohatgi S Soranno DE. Incidence and outcomes of neonatal acute kidney injury (AWAKEN): a multicentre, multinational, observational cohort study. Lancet Child Adolesc Health. (2017) 1:184–94. 10.1016/S2352-4642(17)30069-X 29732396 PMC5933049

[2] Kaddourah A Basu RK Bagshaw SM Goldstein SL AWARE Investigators. Epidemiology of acute kidney injury in critically ill children and young adults. N Engl J Med. (2017) 376:11–20. 10.1056/NEJMoa1611391 27959707 PMC5322803

[3] Hoste EA Bagshaw SM Bellomo R Cely CM Colman R Cruz DN. Epidemiology of acute kidney injury in critically ill patients: the multinational AKI-EPI study. Intensive Care Med. (2015) 41:1411–23. 10.1007/s00134-015-3934-7 26162677

[4] Askenazi DJ Morgan C Goldstein SL Selewski DT Moxey-Mims MM Kimmel PL. Strategies to improve the understanding of long-term renal consequences after neonatal acute kidney injury. Pediatr Res. (2016) 79:502–8. 10.1038/pr.2015.241 26595535 PMC9677947

[5] Zappitelli M Ambalavanan N Askenazi DJ Moxey-Mims MM Kimmel PL Star RA. Developing a neonatal acute kidney injury research definition: a report from the NIDDK neonatal AKI workshop. Pediatr Res. (2017) 82:569–73. 10.1038/pr.2017.136 28604760 PMC9673450

[6] Askenazi DJ Griffin R McGwin G Carlo W Ambalavanan N. Acute kidney injury is independently associated with mortality in very low birthweight infants: a matched case-control analysis. Pediatr Nephrol. (2009) 24:991–7. 10.1007/s00467-009-1133-x 19238451

[7] Koralkar R Ambalavanan N Levitan EB McGwin G Goldstein S Askenazi D. Acute kidney injury reduces survival in very low birth weight infants. Pediatr Res. (2011) 69:354–8. 10.1203/PDR.0b013e31820b95ca 21178824

[8] Viswanathan S Manyam B Azhibekov T Mhanna MJ. Risk factors associated with acute kidney injury in extremely low birth weight (ELBW) infants. Pediatr Nephrol. (2012) 27:303–11. 10.1007/s00467-011-1977-8 21809002

[9] Carmody JB Swanson JR Rhone ET Charlton JR. Recognition and reporting of AKI in very low birth weight infants. Clin J Am Soc Nephrol. (2014) 9:2036–43. 10.2215/CJN.05190514 25280497 PMC4255405

[10] Stojanovic V Barisic N Milanovic B Doronjski A. Acute kidney injury in preterm infants admitted to a neonatal intensive care unit. Pediatr Nephrol. (2014) 29:2213–20. 10.1007/s00467-014-2837-0 24839217

[11] Nagaraj N Berwal PK Srinivas A Berwal A. A study of acute kidney injury in hospitalized preterm neonates in NICU. J Neonatal Perinatal Med. (2016) 9:417–21. 10.3233/NPM-161614 28009331

[12] Sarkar S Askenazi DJ Jordan BK Bhagat I Bapuraj JR Dechert RE. Relationship between acute kidney injury and brain MRI findings in asphyxiated newborns after therapeutic hypothermia. Pediatr Res. (2014) 75:431–5. 10.1038/pr.2013.230 24296799

[13] Askenazi DJ Koralkar R Hundley HE Montesanti A Patil N Ambalavanan N. Fluid overload and mortality are associated with acute kidney injury in sick near-term/term neonate. Pediatr Nephrol. (2013) 28:661–6. 10.1007/s00467-012-2369-4 23224224 PMC5545796

[14] Selewski DT Jordan BK Askenazi DJ Dechert RE Sarkar S. Acute kidney injury in asphyxiated newborns treated with therapeutic hypothermia. J Pediatr. (2013) 162:725–9.e1. 10.1016/j.jpeds.2012.10.002 23149172

[15] Alaro D Bashir A Musoke R Wanaiana L. Prevalence and outcomes of acute kidney injury in term neonates with perinatal asphyxia. Afr Health Sci. (2014) 14:682–8. 10.4314/ahs.v14i3.26 25352889 PMC4209658

[16] Askenazi DJ Ambalavanan N Hamilton K Cutter G Laney D Kaslow R. Acute kidney injury and renal replacement therapy independently predict mortality in neonatal and pediatric noncardiac patients on extracorporeal membrane oxygenation. Pediatr Crit Care Med. (2011) 12:e1–6. 10.1097/PCC.0b013e3181d8e348 20351617

[17] Gadepalli SK Selewski DT Drongowski RA Mychaliska GB. Acute kidney injury in congenital diaphragmatic hernia requiring extracorporeal life support: an insidious problem. J Pediatr Surg. (2011) 46:630–5. 10.1016/j.jpedsurg.2010.11.031 21496529

[18] Fleming GM Sahay R Zappitelli M King E Askenazi DJ Bridges BC. The incidence of acute kidney injury and its effect on neonatal and pediatric extracorporeal membrane oxygenation outcomes: a multicenter report from the kidney intervention during extracorporeal membrane oxygenation study group. Pediatr Crit Care Med. (2016) 17:1157–69. 10.1097/PCC.0000000000000970 27755398 PMC5138084

[19] Aydin SI Seiden HS Blaufox AD Parnell VA Choudhury T Punnoose A. Acute kidney injury after surgery for congenital heart disease. Ann Thorac Surg. (2012) 94:1589–95. 10.1016/j.athoracsur.2012.06.050 22884599

[20] dos Santos El Halal MG Carvalho PR Acute kidney injury according to pediatric RIFLE criteria is associated with negative outcomes after heart surgery in children. Pediatr Nephrol. (2013) 28:1307–14. 10.1007/s00467-013-2495-7 23695031

[21] Taylor ML Carmona F Thiagarajan RR Westgate L Ferguson MA del Nido PJ. Mild postoperative acute kidney injury and outcomes after surgery for congenital heart disease. J Thorac Cardiovasc Surg. (2013) 146:146–52. 10.1016/j.jtcvs.2012.09.008 23040323

[22] Wong JH Selewski DT Yu S Leopold KE Roberts KH Donohue JE. Severe acute kidney injury following stage 1 norwood palliation: effect on outcomes and risk of severe acute kidney injury at subsequent surgical stages. Pediatr Crit Care Med. (2016) 17:615–23. 10.1097/PCC.0000000000000734 27099973

[23] Jetton JG Guillet R Askenazi DJ Dill L Jacobs J Kent AL. Assessment of worldwide acute kidney injury epidemiology in neonates: design of a retrospective cohort study. Front Pediatr. (2016) 4:68. 10.3389/fped.2016.00068 27486571 PMC4950470

[24] Charlton JR Boohaker L Askenazi D Brophy PD D'Angio C Fuloria M. Incidence and risk factors of early onset neonatal AKI. Clin J Am Soc Nephrol. (2019) 14:184–95. 10.2215/CJN.03670318 31738181 PMC6390916

[25] Charlton JR Boohaker L Askenazi D Brophy PD Fuloria M Gien J. Late onset neonatal acute kidney injury: results from the AWAKEN study. Pediatr Res. (2019) 85:339–48. 10.1038/s41390-018-0255-x 30546043 PMC6438709

[26] Selewski DT Gist KM Nathan AT Goldstein SL Boohaker LJ Akcan-Arikan A. The impact of fluid balance on outcomes in premature neonates: a report from the AWAKEN study group. Pediatr Res. (2019). 10.1038/s41390-019-0579-1 31537009 PMC7036003

[27] Selewski DT Akcan-Arikan A Bonachea EM Gist KM Goldstein SL Hanna M. The impact of fluid balance on outcomes in critically ill near-term/term neonates: a report from the AWAKEN study group. Pediatr Res. (2019) 85:79–85. 10.1038/s41390-018-0183-9 30237572 PMC6941736

[28] Askenazi D Abitbol C Boohaker L Griffin R Raina R Dower J. Optimizing the AKI definition during first postnatal week using Assessment of Worldwide Acute Kidney Injury Epidemiology in Neonates (AWAKEN) cohort. Pediatr Res. (2019) 85:329–38. 10.1038/s41390-018-0249-8 30643188 PMC6377843

[29] Harer MW Askenazi DJ Boohaker LJ Carmody JB Griffin RL Guillet R. association between early caffeine citrate administration and risk of acute kidney injury in preterm neonates: results from the AWAKEN study. JAMA Pediatr. (2018) 172:e180322. 10.1001/jamapediatrics.2018.0322 29610830 PMC6137530

[30] Kraut EJ Boohaker LJ Askenazi DJ Fletcher J Kent AL Neonatal Kidney C. Incidence of neonatal hypertension from a large multicenter study [Assessment of Worldwide Acute Kidney Injury Epidemiology in Neonates-AWAKEN]. Pediatr Res. (2018) 84:279–289. 10.1038/s41390-018-0018-8 29795211

[31] Kirkley MJ Boohaker L Griffin R Soranno DE Gien J Askenazi D Acute kidney injury in neonatal encephalopathy: an evaluation of the AWAKEN database. Pediatr Nephrol. (2019) 34:169–76. 10.1007/s00467-018-4068-2 30155763 PMC6986688

[32] Stoops C Boohaker L Sims B Griffin R Selewski DT Askenazi D. The association of intraventricular hemorrhage and acute kidney injury in premature infants from the Assessment of the Worldwide Acute Kidney Injury Epidemiology in Neonates (AWAKEN) study. Neonatology. (2019) 116:321–30. 10.1159/000501708 31461717 PMC6881521

[33] Starr MC Boohaker L Eldredge LC Menon S Griffin R Mayock DE. Acute kidney injury and bronchopulmonary dysplasia in premature neonates born less than 32 weeks' gestation. Am J Perinatol. (2019). 10.1055/s-0039-3400311 31777046 PMC7409513

[34] Starr MC Boohaker L Eldredge LC Menon S Griffin R Mayock D. Acute kidney injury is associated with poor lung outcomes in infants born >/=32 weeks of gestational age. Am J Perinatol. (2020) 37:231–40. 10.1055/s-0039-1698836 31739364 PMC7408289

