# Supplementary material for: AWAKEN-Ing a New Frontier in Neonatal Nephrology
Source: Front Pediatr. 2020 Feb 7;8:21. doi: 10.3389/fped.2020.00021 (PMC7025457; doi:10.3389/fped.2020.00021)
Supplement: Supplementary file 1 [file Presentation_1.PPTX]

## Slide 1
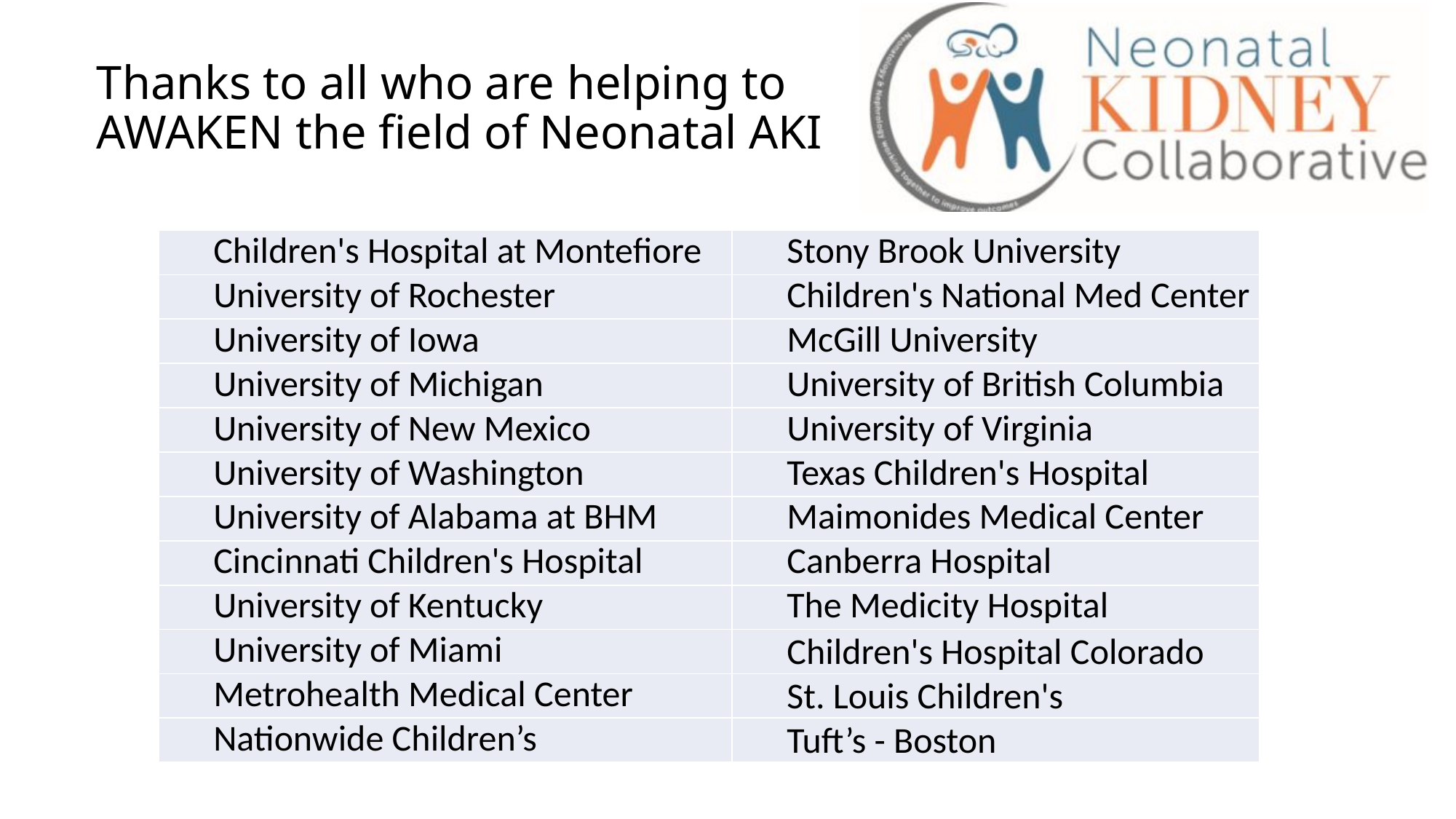

# Thanks to all who are helping to AWAKEN the field of Neonatal AKI
| Children's Hospital at Montefiore | Stony Brook University |
| --- | --- |
| University of Rochester | Children's National Med Center |
| University of Iowa | McGill University |
| University of Michigan | University of British Columbia |
| University of New Mexico | University of Virginia |
| University of Washington | Texas Children's Hospital |
| University of Alabama at BHM | Maimonides Medical Center |
| Cincinnati Children's Hospital | Canberra Hospital |
| University of Kentucky | The Medicity Hospital |
| University of Miami | Children's Hospital Colorado |
| Metrohealth Medical Center | St. Louis Children's |
| Nationwide Children’s | Tuft’s - Boston |

## Slide 2
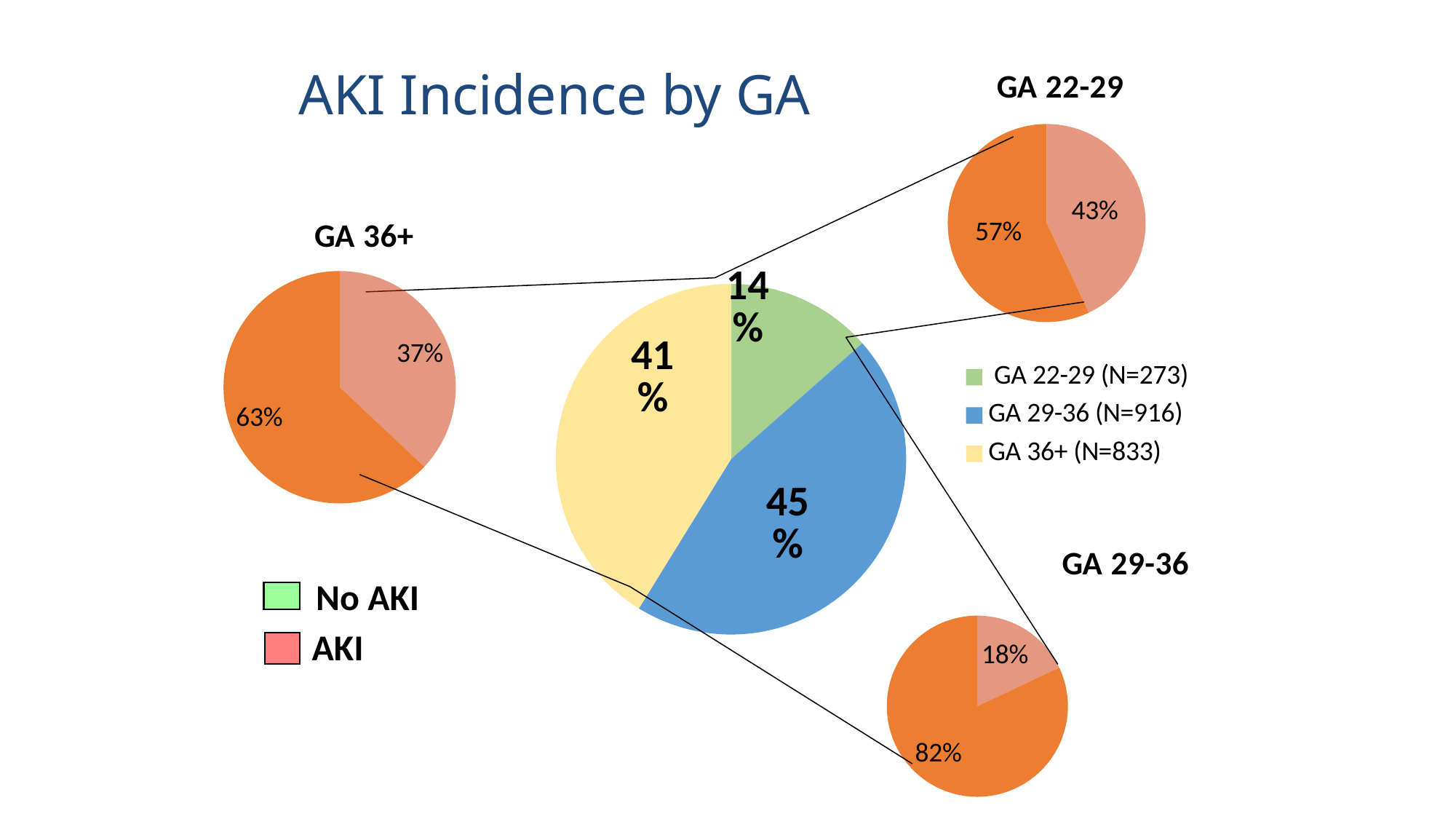

AKI Incidence by GA
### Chart: GA 22-29
| Category | AKI Percentage GA 22-29 |
|---|---|
| AKI (N=114) | 0.43 |
| No AKI (N=159) | 0.57 |
### Chart: GA 36+
| Category | AKI Percentage GA 36+ |
|---|---|
| AKI (N=297) | 0.37 |
| No AKI (N=536) | 0.63 |
### Chart
| Category | Percentage of Cohort |
|---|---|
| GA 22-29 (N=273) | 0.135 |
| GA 29-36 (N=916) | 0.453 |
| GA 36+ (N=833) | 0.412 |
### Chart: GA 29-36
| Category | AKI Percentage GA 29-36 |
|---|---|
| AKI (N=156) | 0.18 |
| No AKI (N=760) | 0.82 |No AKI
AKI

## Slide 3
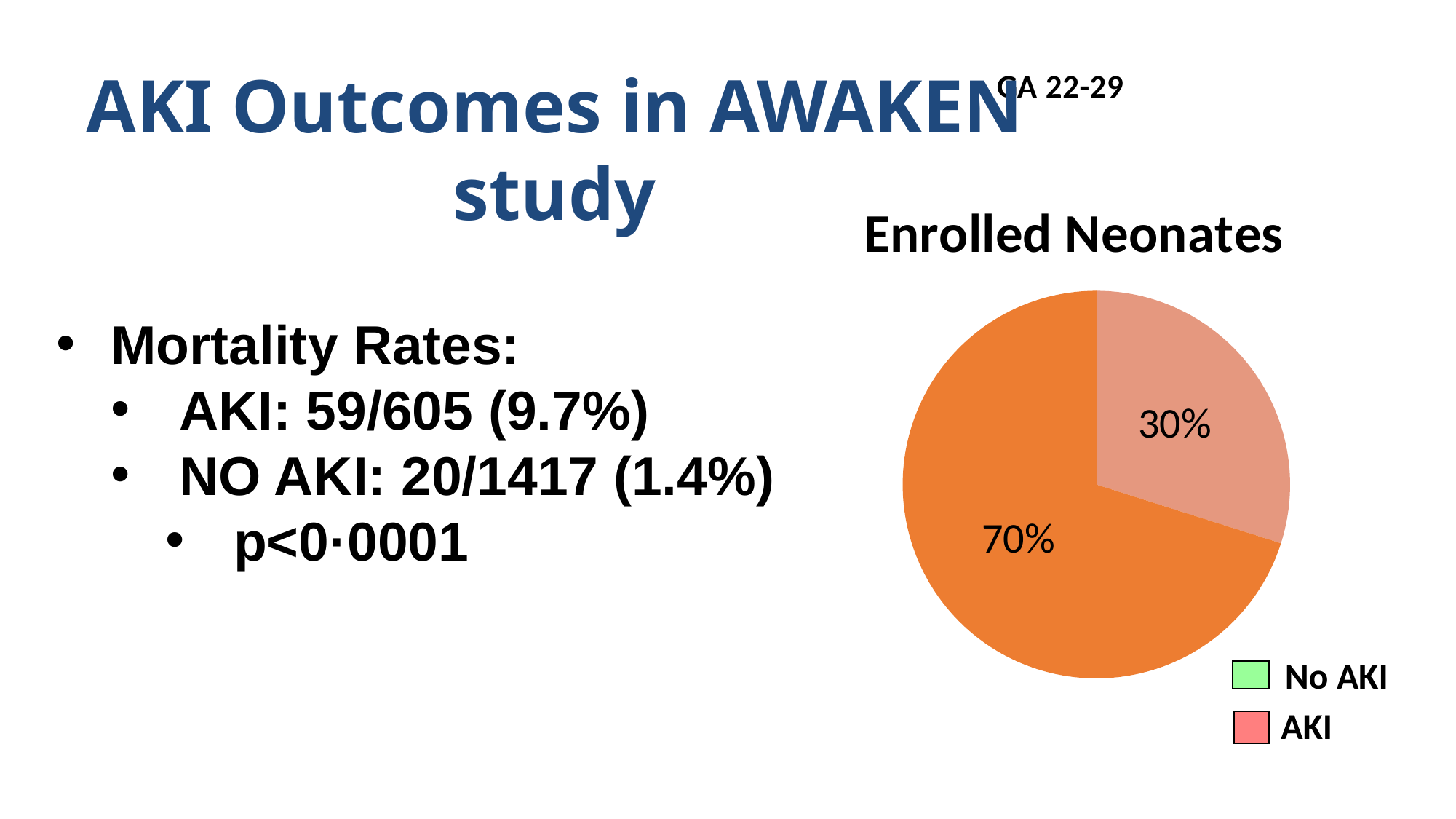

AKI Outcomes in AWAKEN study
### Chart: GA 22-29
| Category |
|---|
### Chart: Enrolled Neonates
| Category | AKI Percentage GA 22-29 |
|---|---|
| AKI (N=114) | 0.299 |
| No AKI (N=159) | 0.701 |Mortality Rates:
AKI: 59/605 (9.7%)
NO AKI: 20/1417 (1.4%)
p<0·0001
No AKI
AKI

## Slide 4
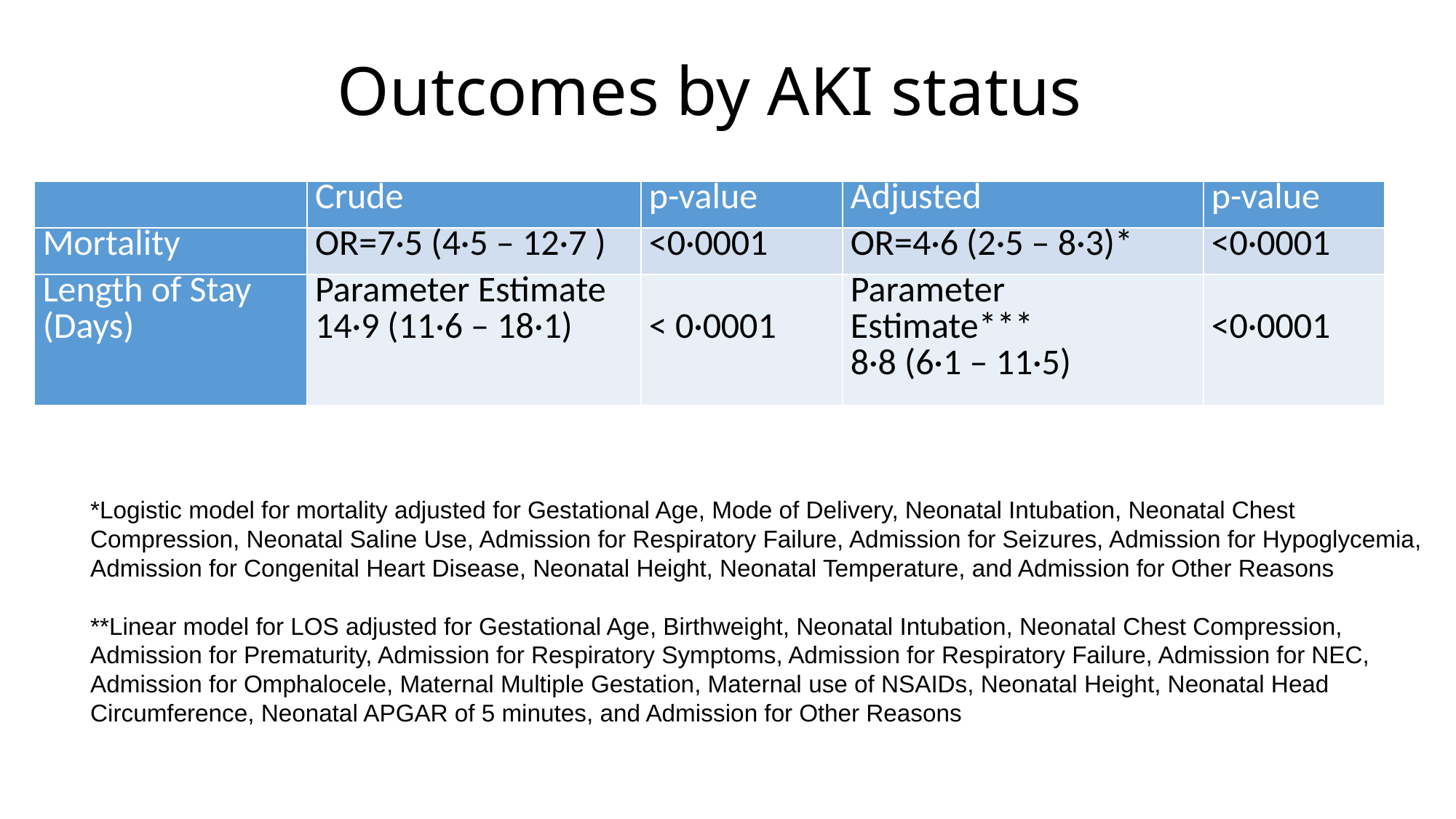

# Outcomes by AKI status
| | Crude | p-value | Adjusted | p-value |
| --- | --- | --- | --- | --- |
| Mortality | OR=7·5 (4·5 – 12·7 ) | <0·0001 | OR=4·6 (2·5 – 8·3)\* | <0·0001 |
| Length of Stay (Days) | Parameter Estimate 14·9 (11·6 – 18·1) | < 0·0001 | Parameter Estimate\*\*\* 8·8 (6·1 – 11·5) | <0·0001 |
*Logistic model for mortality adjusted for Gestational Age, Mode of Delivery, Neonatal Intubation, Neonatal Chest Compression, Neonatal Saline Use, Admission for Respiratory Failure, Admission for Seizures, Admission for Hypoglycemia, Admission for Congenital Heart Disease, Neonatal Height, Neonatal Temperature, and Admission for Other Reasons
**Linear model for LOS adjusted for Gestational Age, Birthweight, Neonatal Intubation, Neonatal Chest Compression, Admission for Prematurity, Admission for Respiratory Symptoms, Admission for Respiratory Failure, Admission for NEC, Admission for Omphalocele, Maternal Multiple Gestation, Maternal use of NSAIDs, Neonatal Height, Neonatal Head Circumference, Neonatal APGAR of 5 minutes, and Admission for Other Reasons
